# The Effects of Fluency Enhancing Conditions on Sensorimotor Control of Speech in Typically Fluent Speakers: An EEG Mu Rhythm Study

**DOI:** 10.3389/fnhum.2018.00126

**Published:** 2018-04-04

**Authors:** Tiffani Kittilstved, Kevin J. Reilly, Ashley W. Harkrider, Devin Casenhiser, David Thornton, David E. Jenson, Tricia Hedinger, Andrew L. Bowers, Tim Saltuklaroglu

**Affiliations:** ^1^Department of Audiology and Speech Pathology, The University of Tennessee Health Science Center, Knoxville, TN, United States; ^2^Department of Communication Disorders, The University of Arkansas, Fayetteville, AR, United States

**Keywords:** speech production, fluency enhancing conditions, EEG, mu rhythm, independent component analysis

## Abstract

**Objective**: To determine whether changes in sensorimotor control resulting from speaking conditions that induce fluency in people who stutter (PWS) can be measured using electroencephalographic (EEG) mu rhythms in neurotypical speakers.

**Methods**: Non-stuttering (NS) adults spoke in one control condition (solo speaking) and four experimental conditions (choral speech, delayed auditory feedback (DAF), prolonged speech and pseudostuttering). Independent component analysis (ICA) was used to identify sensorimotor μ components from EEG recordings. Time-frequency analyses measured μ-alpha (8–13 Hz) and μ-beta (15–25 Hz) event-related synchronization (ERS) and desynchronization (ERD) during each speech condition.

**Results**: 19/24 participants contributed μ components. Relative to the control condition, the choral and DAF conditions elicited increases in μ-alpha ERD in the right hemisphere. In the pseudostuttering condition, increases in μ-beta ERD were observed in the left hemisphere. No differences were present between the prolonged speech and control conditions.

**Conclusions**: Differences observed in the experimental conditions are thought to reflect sensorimotor control changes. Increases in right hemisphere μ-alpha ERD likely reflect increased reliance on auditory information, including auditory feedback, during the choral and DAF conditions. In the left hemisphere, increases in μ-beta ERD during pseudostuttering may have resulted from the different movement characteristics of this task compared with the solo speaking task. Relationships to findings in stuttering are discussed.

**Significance**: Changes in sensorimotor control related feedforward and feedback control in fluency-enhancing speech manipulations can be measured using time-frequency decompositions of EEG μ rhythms in neurotypical speakers. This quiet, non-invasive, and temporally sensitive technique may be applied to learn more about normal sensorimotor control and fluency enhancement in PWS.

## Introduction

Sensorimotor control for speech production is achieved via the integration of feedback and feedforward control mechanisms (Houde and Jordan, 1998; Jones and Munhall, 2005; Purcell and Munhall, 2006; Bauer et al., 2007). Feedforward control is associated with the activation of speech motor programs in ventral premotor areas in the left frontal lobe. Projections from the premotor area to bilateral primary motor cortex encode motor commands for producing the sound or syllable of the active motor program. Activation of a motor program also engages feedback controllers for speech via projections to auditory and somatosensory association areas. These projections encode forward models that transform the current motor commands into the desired or expected sensory outcomes. Comparisons between desired and actual feedback allow for monitoring the accuracy of speech output. Discrepancies between these signals generate an error signal, the magnitude and direction of which are mapped onto corrective motor commands via projections from auditory and somatosensory areas to frontal speech motor areas. The projections encode a kind of inverse model that transforms the sensory error into a corrective motor response. While feedforward control for speech is primarily a left hemisphere dominant function (Ghosh et al., 2008; Peeva et al., 2010), feedback control for speech exhibits a right hemisphere dominance (Riecker et al., 2000; Toyomura et al., 2007; Tourville et al., 2008; Golfinopoulos et al., 2011; Tourville and Guenther, 2011; Niziolek and Guenther, 2013). A similar hemispheric differentiation between feedforward and feedback control has been identified for limb movements (Grafton et al., 2008).

These accounts of feedforward and feedback control in healthy speakers also have provided insight into sensorimotor speech disorders. For example, stuttering is associated with deficits in both inverse (Civier et al., 2010; Cai et al., 2012) and forward models (Daliri et al., 2014; Daliri and Max, 2015). For example, the findings of several studies indicate that stuttering is related to impaired feedforward control, related to either readout of feedforward motor programs (Civier et al., 2013) or inverse mapping of auditory states onto motor commands (Cai et al., 2012). Deficits in feedforward control in stuttering are consistent with the finding of speech-related hypoactivation in left premotor regions (Watkins et al., 2008; Chang et al., 2009; Kell et al., 2009; Loucks et al., 2011; Toyomura et al., 2011) and, as a consequence, give rise to an over-reliance on auditory feedback as indicated by the increased right hemisphere activity during speech in persons who stutter (PWS; Fox et al., 1996, 2000; Braun et al., 1997; De Nil et al., 2000; Ingham, 2001; Brown et al., 2005; Sakai et al., 2009). In addition, other studies have reported evidence of deficits in forward, motor-to-sensory transformations (Daliri et al., 2014; Daliri and Max, 2015) that are critical to error detection and correction in feedback control of speech (Tourville et al., 2008; Tourville and Guenther, 2011).

Models of intact sensorimotor control provide important tools for advancing understanding of speech disorders. Similarly, the patterns of neural activity associated with these disorders have the potential to inform models of speech sensorimotor control (Kent et al., 2000). An aspect of stuttering with implications for the neural control of fluent speech involves techniques that enhance speech fluency in PWS by altering sensorimotor control. Fluency enhancing techniques, or conditions, may be classified as exogenous or endogenous (Kalinowski and Saltuklaroglu, 2003). Exogenous conditions enhance fluency by means of an external (typically auditory) sensory signal, while endogenous conditions operate by exerting volitional changes in speech motor control. Choral speech (speaking in unison) and delayed auditory feedback (DAF) are two exogenous conditions that have been shown to induce fluency in PWS. In choral speech, fluency enhancement is achieved relatively effortlessly and requires no training. Stuttering is immediately reduced by 90%–100% (Cherry and Sayers, 1956; Andrews et al., 1982). DAF manipulates auditory feedback by playing the speaker’s voice back to them with an exogenously derived temporal delay. It has been shown to reduce the frequency of stuttering behaviors in PWS by 50%–80% (Goldiamond, 1965; Andrews et al., 1983; Kalinowski et al., 1993). Though highly effective, one difficulty that typically cannot be overcome by exogenous fluency enhancing signals is the preponderance of stuttering during speech initiation (Brown, 1938; Saltuklaroglu et al., 2009). Initiation is thought to be particularly difficult in PWS because weak forward models instantiated prior to receiving auditory feedback are more susceptible to error detection (Guenther et al., 2006). Saltuklaroglu et al. (2009) attempted to compensate for the initiation difficulties by introducing an exogenous carrier phrase prior to choral speech. They reported an overall 98% stuttering reduction compared to a control condition. Importantly, almost no stuttering was observed during initiation, suggesting that the additional exogenous feedback helped overcome initiation difficulties, possibly because the carrier phrase primed the sensorimotor system to initiate speech (Saltuklaroglu et al., 2009).

Endogenous fluency enhancing conditions include prolonged speech and pseudostuttering. Prolonged speech is a commonly used and effective therapeutic technique (O’Brian et al., 2003) that requires “stretching out” voiced phonemes, which consequently can reduce speech rates to as low as 30–60 syllables per minute (Ingham and Andrews, 1973). Pseudostuttering is an adaptation of an early technique called “bouncing” (Johnson, 1959). It is an endogenous therapeutic technique requiring multiple volitional repetitions of the first syllable in an utterance. For example, the phrase “my name is….” would begin with “Muh muh muh my…” It has been found to increase fluency in the ensuing utterance (Saltuklaroglu et al., 2004). Though it is not clear how this technique enhances fluency, it may be likened to an endogenous form of “shadow speech”. Shadow speech is an exogenous condition requiring a second speaker that possesses powerful fluency enhancing effects similar to those of choral speech (Cherry, 1953; Andrews et al., 1982). However, rather than speaking in unison, shadowing requires a PWS to immediately imitate what the speaker said.

Changes in frontal sensorimotor activity resulting from the use of both exogenous and endogenous techniques have been measured in both PWS and non-stuttering (NS) cohorts. In PWS, choral speech has been found in some studies to “normalize” neural function by reducing right hemisphere frontal activity (Fox et al., 1996, 2000). In contrast, choral speech in NS has not been found to produce remarkable changes in frontal activity (Toyomura et al., 2011). DAF has been found to induce increases in right frontal activity in both PWS and NS (Watkins et al., 2008; Sakai et al., 2009; Toyomura et al., 2011), suggesting an increased contribution from feedback control circuits. The similar effect of DAF on neural activity in PWS and NS contrasts somewhat with the differential effects of choral speech on PWS and NS and with earlier studies of DAF reporting paradoxical effects in PWS and NS. Specifically, although DAF is fluency enhancing in PWS, it has been found to induce disfluency in NS speakers, especially when longer delays are introduced (i.e., 200 ms; Stuart et al., 2002; Corey and Cuddapah, 2008). Thus, further investigation of changes in sensorimotor control via exogenous signals is warranted.

The effects of endogenous fluency enhancement on neural activity also have been investigated. De Nil et al. (2008) demonstrated that both prolonged speech and pseudostuttering increase right hemisphere activity in PWS, but produce non-significant changes in neural activity in NS. However, following completion of therapy using prolonged speech, a reduction in right hemisphere activity also has been observed in PWS (De Nil et al., 2003; Neumann et al., 2005). Thus, the immediate effects of prolonged speech may be explained by increased time allowed for speech processing (van Lieshout et al., 1996; Max et al., 2004). Moreover, long-term fluency-enhancing effects might reflect more normalized feedforward control. Considering the differences in the short vs. long-term effects of endogenous techniques in PWS and the differences between the PWS and NS, changes in sensorimotor control related to endogenous speech manipulations also merit further investigation.

Previous findings indicate that fluency-enhancing conditions alter aspects of sensorimotor control in PWS related to the control deficits during stuttering. Moreover, these conditions also elicit neural changes in NS that are often distinct from those observed in PWS. Many fluency-enhancing effects on sensorimotor processing in NS are difficult to explain with existing models of speech motor control and, as such, their causal mechanism and incorporation into existing models of speech production are important areas of investigation.

To summarize, few studies have investigated how these fluency-enhancing conditions alter sensorimotor control in NS populations. Understanding the neural mechanisms underlying these conditions from the perspective of normal motor control will inform current theoretical models and allow for critical comparisons to data from PWS. As such, the present study evaluated neural sensorimotor processing during endogenous and exogenous fluency-enhancing condition in NS. One way to measure differences in sensorimotor control in fluency enhancing conditions is to use temporally sensitive measures of changes in the electroencephalographic (EEG) mu (μ) rhythm power. EEG measures of μ rhythm activity have traditionally been obtained from alpha band (8–13 Hz) activity over the sensorimotor cortex (for a review see Fox et al., 2016). However, since 1989 (Tiihonen et al., 1989) magnetoencephalographic (MEG) studies have revealed unified mu rhythms, with both alpha and beta (15–25 Hz) spectral peaks, that can be localized to a single dipole emanating from PMC/primary motor regions (Hari and Salmelin, 1997; Pfurtscheller et al., 1997; Crone et al., 1998; Szurhaj et al., 2003; Jones et al., 2009).

Similar mu rhythms are now identifiable using independent component analyses (ICA) of raw EEG data (Wang and Jung, 2012; Bowers et al., 2013; Jenson et al., 2014; Cuellar et al., 2016). ICA is particularly useful when applied to EEG data from movement studies. Its ability to blindly separate sources of oscillatory activity allows sensorimotor or other neural activity to be temporally referenced to muscle movements using time-frequency decompositions (Jenson et al., 2014). While oscillatory activity from alpha and beta bands of the mu rhythm (henceforth μ-alpha and μ-beta) are often strongly correlated (Carlqvist et al., 2005; de Lange et al., 2008), they have also been known to dissociate (Brinkman et al., 2014; Jenson et al., 2014) indicating that they reflect distinct yet related sensorimotor functions. In movement tasks, μ-beta suppression is typically linked to motor activity and μ-alpha suppression to a somatosensory response (Hari, 2006).

A characteristic pattern of power suppression is observed in both frequency bands as a muscle begins to contract. Suppression in both alpha and beta bands grows stronger during movement (Pfurtscheller and Neuper, 1992). μ-beta suppression is relatively stable during movement, and is independent of muscle force (Kilavik et al., 2013). In addition, beta suppression is observed prior to movement and beta enhancement (rebound) is observed after a movement (Kilavik et al., 2013). Therefore, changes in μ-beta power are thought to be related to contributions from sensorimotor forward models (Tan et al., 2014; Moisello et al., 2015; Mersov et al., 2016). Similarly, in addition to providing a primary somatosensory response, μ-alpha activity is sensitive to changes in somatosensory (Cheyne et al., 2003), auditory (Tamura et al., 2012), and visual (de Graaf et al., 2013) feedback, perhaps encoding inverse models (Sebastiani et al., 2014). Exploiting the temporal resolution of EEG, time-frequency changes are measured via event-related spectral perturbations (ERSP), which reveal patterns of event-related synchronization (ERS) or deysnchronization (ERD; Makeig, 1993; Pfurtscheller and Lopes da Silva, 1999). ERS and ERD are suggestive of neural inhibition and excitation, respectively. Together, the response properties of μ-alpha and μ-beta strongly suggest that the μ rhythm oscillations can index sensorimotor control across movement tasks, including speech production (Jenson et al., 2014, 2015).

Considering that all of the fluency enhancing conditions discussed alter the way speech is perceived and/or produced, alterations in sensorimotor dynamics are likely to be revealed in μ-alpha and μ-beta oscillations across speaking events. Thus, the goal of the current study is to better understand how exogenous and endogenous speech manipulations alter sensorimotor control in NS. Providing temporally sensitive measures will enhance understanding of how sensorimotor control can be manipulated and data may serve as a basis for future comparisons to PWS under similar conditions. To accomplish the goal, ICA and ERSP are used to identify and temporally decompose μ rhythms from raw EEG data and isolate electromyography (EMG) activity to be used for temporal alignment of neural and muscular data. It is hypothesized that, compared to a control condition, experimental conditions will produce altered μ-alpha and μ-beta desynchronization throughout the time course of speaking events, reflecting changes to sensorimotor control as a result of different speaking conditions. Since speech behaviors during exogenous conditions are cued by external auditory signals, it is predicted that these conditions will most strongly increase reliance on auditory feedback control and consequently, right hemisphere activity (Tourville and Guenther, 2011). Endogenous conditions might be expected to strengthen left hemisphere activity especially in the beta band due to their purported influences on forward modeling. Additionally, they also might increase right hemisphere activity because they involve speaking in a less natural and somewhat novel manner that requires auditory monitoring of the output. However, it is not clear if endogenous manipulations are sufficient to elicit significant sensorimotor control changes in NS speakers.

## Materials and Methods

### Participants

Twenty-six adult native English speakers (23 right-handed) were recruited from the University of Tennessee and surrounding Knoxville area. Participants had a mean age of 26 (range 17–46) and reported no history of cognitive, communicative, or attentional disorders. 18/26 participants were males (8 females) and 23/26 identified as being of Caucasian descent. Table 1 shows a breakdown of all subjects and their contributions to μ clusters. Handedness dominance was assessed with the Edinburg Handedness Inventory (Oldfield, 1971). This study was carried out in accordance with the recommendations of University of Tennessee Health Science Center Institutional Review Board with written informed consent from all subjects. All subjects gave written informed consent in accordance with the Declaration of Helsinki. The protocol was approved by the University of Tennessee Health Sciences Center Institutional Review Board.

**Table 1:** Demographics and cluster contributions.

Subject ID #	Sex	Age	Handedness	Cluster contribution
C1	M	26–30	R	L, R
C2	M	26–30	R	L
C3	M	36–40	R	R
C4	F	21–25	R	L
C5	M	21–25	R	L
C6	M	21–25	R	
C7	F	21–25	R	L
C8	M	31–35	R	
C9	F	16–20	R	L, R
C10	M	41–45	R	L
C11	M	21–25	R	L
C12	F	26–30	L	
C13	F	21–25	R	R
C14	M	36–40	R	
C15	F	16–20	R	
C16	M	26–30	R	
C17	F	21–25	R	L
C18	M	21–25	R	L, R
C19	M	26–30	R	L, R
C20	M	16–20	R	R
C21	F	21–25	R	L, R
C22	M	16–20	R	R
C23	M	26–30	R	L
C24	M	21–25	L	
C25	M	16–20	Ambi	L
C26	M	46–50	R	L, R

### Stimuli

Visual stimulus tokens consisting of 9–12 syllable sentence fragments (mean = 10 syllables) were derived from 6th grade reading passages. Tokens were unique across conditions, with each token only being used once per participant. Example tokens include “When you look at mountains they seem permanent”, and “Friends on a cotton plantation near”, and “Had attended thirty seven different”. In the choral condition, synthetic speech recordings using analogs of a human male speaker were made using the Neospeech Text to Speech application. Audacity software version 2.1.0 (Team Audacity, 2015) was used to segment the generated sentences into individual tokens of the synthetic speech analogs for each trial. Using the same process, a carrier phrase, “Hello”, which was 1270 ms in duration, was inserted prior to each individual token.

### Design

A 5-condition within-subject design was employed. The first condition (solo) was used as a control task that provided a baseline of unassisted speaker fluency. The four experimental conditions are known to enhance fluency exogenously (conditions 2 and 3) and endogenously (conditions 4 and 5). Participants were required to:

1. Speak without exogenous or endogenous alteration (solo—control condition).
2. Speak in unison with a recording (choral).
3. Speak under DAF.
4. Speak while prolonging the vowels in each word (prolonged).
5. Speak while pseudostuttering (pseudo).

**Figure 1:**
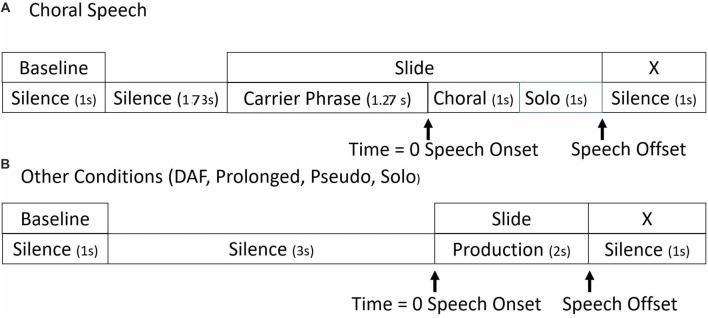
Timelines. Seven-thousand milliseconds epoch timelines for single trials in **(A)** choral and **(B)** all other experimental conditions.

The timelines for stimulus presentation are shown in Figure 1. In all conditions, the entire epoch length was −4000 ms to 3000 ms. Baselines for ERSP analyses were taken from the 1000 ms period beginning at −4000 ms. The stimuli played for 2000 ms, with the cue to speak being 0 ms. At 2000 ms, an “X” appeared on the screen, which was the cue to stop speaking.

There were slight differences in the timelines between the choral and the other speaking conditions. In all conditions except choral, the cue to speak was a visual stimulus presentation of the tokens. In the choral condition, the carrier phrase and the visual presentation of the tokens began at −1270 ms on the timeline. At 0 ms, the choral accompaniment was presented for 1000 ms. The choral accompaniment was matched to the visual stimuli. Participants then spoke without the choral accompaniment for 1000 ms. Therefore, in this condition, participants were able to read what they were about to say while they heard the carrier phrase and then say it with choral accompaniment.

### Procedure

Prior to data collection, the researcher trained each participant on what would be required in the speaking conditions. This included how to respond to the choral signal and to listen to the DAF. It also included training on the endogenous techniques. Participant training for endogenous conditions used reading passages not used as experiment tokens. The researchers required the participants to demonstrate effective use of slow prolongations, producing 30–60 syllables per minute (SPM) by prolonging the vowels of every word. They also demonstrated pseudostuttering by producing three syllable iterations of the initial syllable of a sentence in a controlled manner. Data collection began after participants were clear on the instructions and had demonstrated mastery of the endogenous conditions.

The experiment was conducted in an electronically and magnetically shielded, double-walled, sound-treated booth. Participants were seated in a comfortable reclining armchair with their heads and necks well supported. Compumedics Neuroscan Stim 2 version 4.3.3 software was used to present stimuli to participants via a PC. Visual stimuli appeared on a 69.5 × 39.0 cm display placed 132 cm in front of the reclining chair. All conditions were presented in two blocks of 40 trials each. The order of the 10 blocks (5 conditions × 2 blocks) was randomized for each participant.

In the solo (control) condition, participants were instructed to speak naturally using a normal rate. In the choral condition, participants were instructed to begin speaking as they heard the choral accompaniment immediately after the carrier phrase and continue to speak until the cue to stop. In the DAF conditions, they were instructed to speak while listening to their speech fed back to them with a 100 ms delay. 100 ms was chosen as it is in a range chosen by stuttering clients for promoting fluency (van Borsel et al., 2003), has been used in studies using short utterances (Saltuklaroglu et al., 2009), and was considered short enough not to disrupt speech in NS populations (Stuart et al., 2002). Auditory stimuli were delivered through insert headphones. DAF was generated using Audacity software on a windows laptop. For this condition only, participants were required to speak into a Rode NT1-A condenser microphone held at chest level. In both DAF and choral conditions, insert earphones were used to deliver the auditory signals. In the prolonged speech condition, participants spoke using vowel prolongations that induced a slow speech rate (30–60 SPM). In the pseudostuttering condition, participants produced three repetitions of the initial syllable prior to speaking the remainder of the token.

### EEG Acquisition

Whole-head EEG data were recorded from a 68 channel NeuroScan Quikcap based on the 10–20 extension (Chatrian et al., 1988) of the international standard system (Jasper, 1958). Neural channels were accompanied by two electrocardiogram (EKG) and two EMG channels. Recording channels were re-referenced to the linked mastoid channels (M1, M2) for common mode noise reduction. The electro-oculogram was captured by two pairs of recording electrodes placed above and below the left eye (VEOU, VEOL) as well as on the medial and lateral canthi of the left eye (HEOL, HEOR), which recorded vertical and horizontal eye movements, respectively. The two EMG electrodes were placed above and below the lips in order to capture speech related peri-labial muscle activity (Gracco, 1988).

EEG data were acquired using Compumedics NeuroScan 4.3.3 software coupled with the Synamps 2 system. Data were band pass filtered (0.15–100 Hz) prior to digitization by a 24 bit analog to digital converter with a sampling rate of 500 Hz. Data collection was referenced to the cue to initiate production thus, time zero represents the initiation cue.

### Preprocessing

All EEG data processing was performed in EEGLAB 13.5.4b (Brunner et al., 2013), an open source MATLAB toolbox. Data for each participant were processed at the individual level, then analyzed at both the individual and group levels. Steps performed at each stage are outlined below:

Individual Processing:

1. Preprocessing of 10 raw files for each subject (2 blocks × 5 conditions).
2. ICA of concatenated data files for each participant.
3. Localization estimation via fitting of equivalent current dipole (ECD) models for all neural and non-neural components.

Group Analysis:

1. Similar components across subjects clustered by Principal Component Analysis (PCA) on the basis of commonalities in spectra, scalp maps, and dipole location.
2. Visual inspection of clusters resulting from PCA to identify left and right μ clusters and validate cluster membership. Neighboring clusters were also examined to identify misallocated components.
3. Time-frequency decomposition of left and right μ clusters performed by ERSP analysis.
4. Mean ECD source computed for μ clusters by averaged localization sources of all components within the clusters.

#### Data Preprocessing

Both raw data files for each condition (one per block) were appended to create a single file for each condition per subject. The data were then down sampled from 500 Hz to 256 Hz to reduce the computational demands of further processing steps. All EEG data were referenced to the mastoid channels (M1, M2) for common mode noise reduction. The data were band pass filtered twice (once at the beginning of the preprocessing and once as the final step) between 3 Hz and 34 Hz in order to capture frequency bands of interest and remove non-stereotypical noise. Six second trial epochs (ranging from −4000 ms to 2000 ms around time zero) were then extracted from the continuous data. The resulting data files for each subject, in all conditions, were visually inspected, and all epochs containing gross artifact (>200 μV) were removed from the data. A minimum of 40 usable trials per condition per participant was required for an effective ICA decomposition.

#### Independent Component Analysis (ICA)

Prior to ICA training, pre-processed EEG data for each participant were concatenated across all five conditions so that a single set of ICA weights could be obtained. This allowed for a comparison of activity to be made across conditions within spatially fixed ICs. An extended Infomax algorithm (Lee et al., 1999) was used to decorrelate the data matrix prior to ICA rotation. ICA training was provided using the “extended runica” algorithm in EEGLAB13.5.4b with an initial learning rate set to 0.001 and a stopping weight of 10–7. Following decomposition, 66 ICs were yielded for each participant reflecting the total number of recording electrodes (68–2 reference electrodes, M1 and M2). Scalp maps for each IC were obtained by projecting the inverse weight matrix (W^−1^) back onto the spatial EEG channel configuration.

Following ICA decomposition, ECD models for each IC were computed using the DIPFIT2 toolbox, freely available at https://sccn.ucsd.edu/wiki/A08:_DIPFIT (Oostenveld and Oostendorp, 2002). Standard 10–20 electrode coordinates were warped to a boundary element head model (BEM) based on the MNI152 template (Mazziotta et al., 2001), followed by automated coarse-fitting to yield a single dipole model for each of 1716 ICs (66 ICs × 26 participants). Dipole localization entails back-projecting the signal to a source that may have generated the scalp potential distribution for a given IC, and then computing the best forward model to explain the highest percentage of scalp map variance (Delorme et al., 2012). The discrepancy between this forward model and the original scalp-recorded signal constitutes residual variance (RV), a putative measure of “goodness of fit” where lower values indicate higher confidence in the dipole model.

#### EEGLAB STUDY

Group data analyses were conducted via the EEGLAB STUDY module. The STUDY module allows ICA data from multiple participants across conditions to be analyzed using specified designs. In the current study, there were five paired contrasts of interest (solo vs. each experimental condition). The STUDY module allows further filtering to be applied with respect to the RV in dipole localization and inclusion vs. exclusion of “out-of-head” dipoles. Thus, ICA files with dipole information from each individual (see above) were applied to the two separate STUDY modules. For the purposes of measuring neural activity, only “in-head” dipoles with RV <20% were analyzed.

For the purposes of identifying peri-labial EMG activity, a second STUDY was conducted that included “all” dipoles from in-head and out-of-head. In this second STUDY, the RV criterion was raised to 50% (Gramann et al., 2010), because EMG activity emanates from outside the head and by nature, muscular movement incurs higher unexplained RV.

#### Principal Component Clustering of ICs

The EEGLAB STUDY module was used to perform all group level analyses, as it enables comparison of ICA data across conditions and between groups. ICs were initially preclustered based on similarities in scalp map, spectra, and dipole location. PCA was implemented via the K-means statistical toolbox, allocating the ICs to 40 component clusters from which left and right μ clusters were identified. Final component designation to left and right μ clusters was based largely on the initial results of PCA, though neighboring clusters were visually inspected for misallocated components. Inclusion criteria for the μ clusters were a characteristic μ spectrum, RV < 20%, and ECD localization across the sensorimotor cortex (BA 1, 2, 3, 4, 6). Any components not meeting these criteria were removed from the μ clusters and excluded from further analysis. An additional component cluster, representing peri-labial EMG activity, was identified from the results of PCA in the second study. As myogenic activity is associated with higher RV, membership to the peri-labial EMG cluster was based primarily on dipole location and the presence of ERSP activity.

### Source Localization

ECD dipole locations (generated via DIPFIT2) were submitted to PCA in the STUDY module. Talairach cortical atlas was used to confirm the dipole location, with electrode locations cross-registered between realistic cortical anatomy and standard spherical (BESA) head models (Towle et al., 1993). Source localization for the μ clusters of interest was based on the mean of the Talairach coordinates for each contributing dipole. As localization was based on standard rather than subject-specific head models and channel locations, ECD location best served as confirmation of component origin within sensorimotor regions that are known to generate μ rhythms rather than an exact cortical location (i.e., ventral vs. dorsal premotor cortex). Even without exact locations, the necessary inclusion step of considering the spectral characteristic of each component included in the cluster helped to ensure the sensorimotor nature of the components.

### Component Measures

#### ERSP

ERSP analyses were conducted to quantify fluctuations in spectral power (in normalized dB units) across the trial epochs in the frequency bands of interest. Time-frequency transformations were generated with a Morlet wavelet rising linearly from three cycles at 3 Hz to 25.6 cycles at 34 Hz. Spectral perturbations were referenced to a silent 1000 ms baseline taken from the inter-trial interval. A surrogate distribution was constructed from 200 randomly sampled time points within this baseline period, against which individual ERSP changes were generated with a bootstrap resampling method with a threshold of *p* < 0.05 (with false discovery rate, FDR). Data from all experimental conditions from 5 Hz to 30 Hz and between −500 ms and 2500 ms were included in the ERSP analysis. The random distribution represents the null hypothesis that no conditional differences exist. Type 1 error was controlled by correcting for FDRs (Benjamini and Hochberg, 2000). Statistical analyses used a series of paired comparisons between the solo (control) and each of the four experimental conditions with a 95% confidence interval (*p* < 0.05).

## Results

### Behavioral

For each condition, participants reported that they completed each task accurately, using the previously trained techniques as instructed. Participants also reported no stuttering in the DAF condition. For solo, choral and DAF conditions, participants were easily able to speak all of the 9–12 syllable stimuli. In the prolonged condition, participants were only able to produce the first 1–3 syllables in the allotted 2000 ms speaking window. In the pseudostuttering condition, participants indicated that they could produce the three repetitions and 1–3 additional syllables in the allotted time. Following completion, all participants reported that they produced slow prolongations and pseudostuttering in the same manner that they were trained. Data from EMG recordings also support the claim that participants performed endogenous tasks accurately, as EMG activity began shortly following the cue to speak.

### μ and EMG Cluster Characteristics

**Figure 2:**
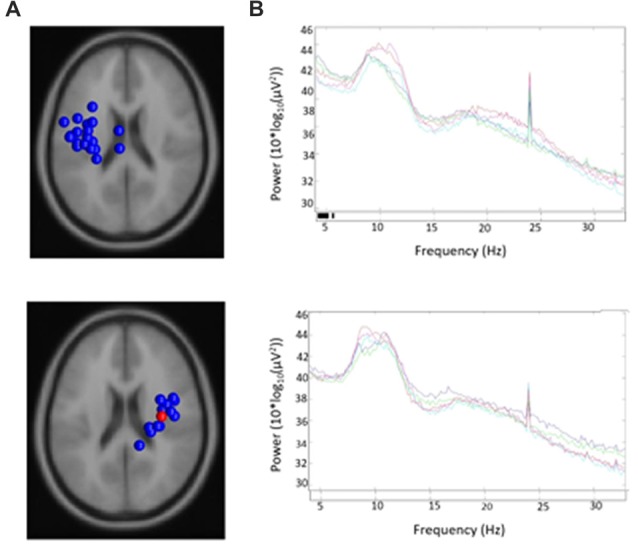
Sources and spectra. Equivalent current dipole (ECD) sources and average spectra for each condition in **(A)** left and **(B)** right clusters of mu components.

19/24 participants were right-handed (two contributing participants were left-handed and were excluded to control for potential differences in cerebral dominance for language) and produced components with <20% unexplained RV that contributed to at least one μ cluster. Table 1 describes participants and their μ cluster contributions. For the left μ cluster, the Talaraich ECD location was [−35, −10, 46] which contained 21 ICs and localized to BA 6. The Talaraich ECD location for the right was [32, −11, 45] which contained 12 ICs and localized to BA 6. The average RV in the μ clusters was 6.05% and 4.31% for the left and right clusters, respectively. Of the 19 participants who contributed to the μ clusters, 14/19 contributed to the EMG cluster. This cluster had an average unexplained RV of 8.06%. While all participants had an EMG component, some did not meet inclusion criteria, primarily due to location. Figure 2 displays the spectra and dipole clusters for the left and right μ clusters, respectively.

### Time Frequency Analyses

**Figure 3:**
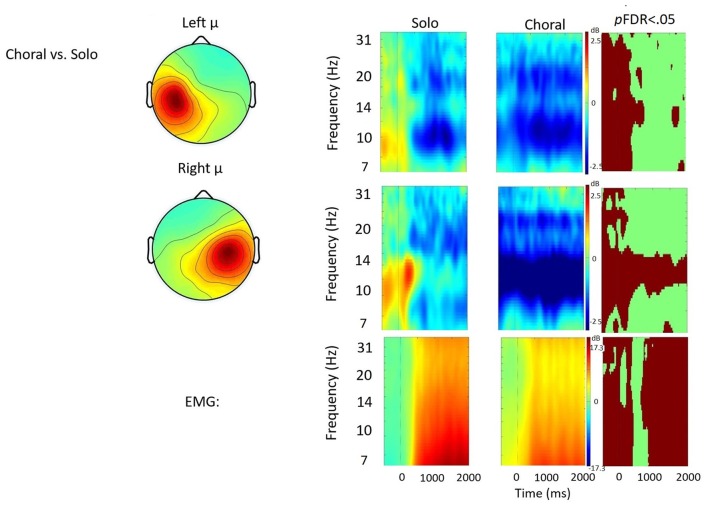
Solo vs. choral contrast. Event-related spectral perturbations (ERSP) analyses showing time-frequency differences between the solo and choral condition. The first two rows show left and right mu rhythm scalp maps, followed by time-frequency patterns of event-related synchronization (ERS) and event-related desynchronization (ERD), followed by statistically different (*p*FDR < 0.05) time frequency voxels between the two conditions. The last row shows the differences in EMG activity.

**Figure 4:**
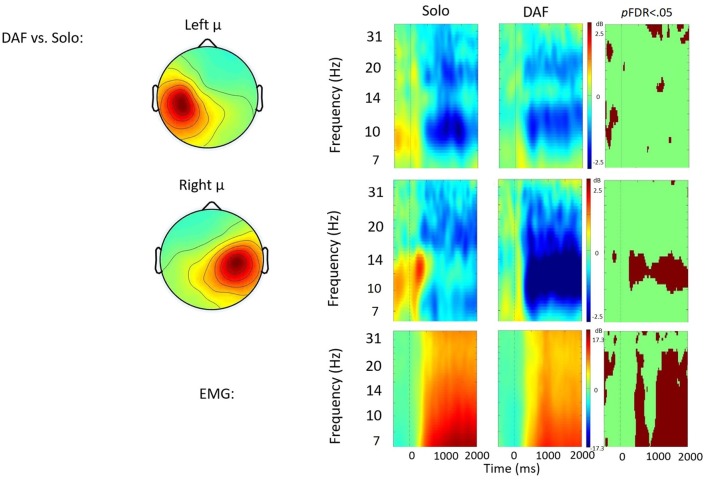
Solo vs. delayed auditory feedback (DAF) contrast. ERSP analyses showing time-frequency differences between the solo and DAF condition. The first two rows show left and right mu rhythm scalp maps, followed by time-frequency patterns of ERS and ERD, followed by statistically different (*p*FDR < 0.05) time frequency voxels between the two conditions. The last row shows the differences in EMG activity.

**Figure 5:**
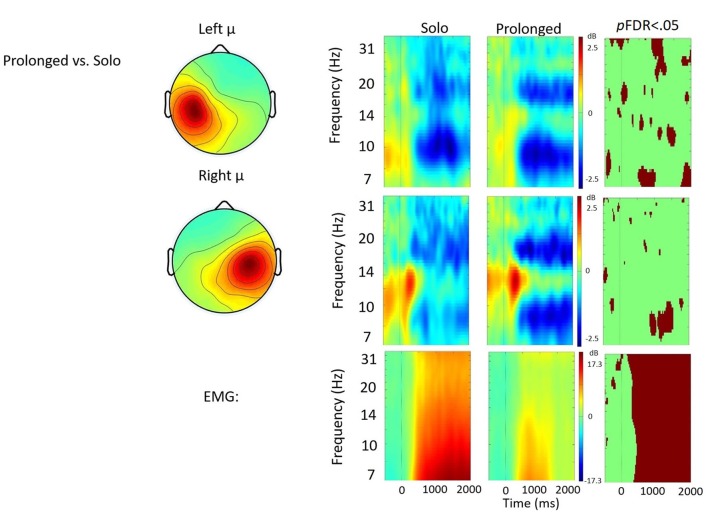
Solo vs. prolonged speech contrast. ERSP analyses showing time-frequency differences between the solo and prolonged speech condition. The first two rows show left and right mu rhythm scalp maps, followed by time-frequency patterns of ERS and ERD, followed by statistically different (*p*FDR < 0.05) time frequency voxels between the two conditions. The last row shows the differences in EMG activity.

**Figure 6:**
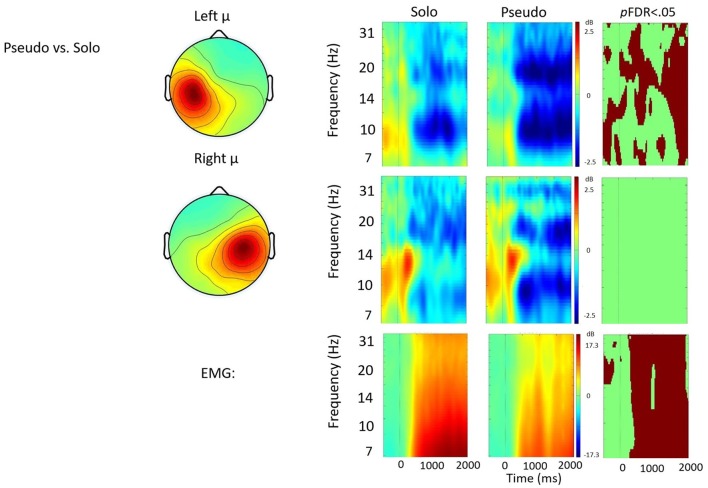
Solo vs. pseudostuttering contrast. ERSP analyses showing time-frequency differences between the solo and pseudostuttering condition. The first two rows show left and right mu rhythm scalp maps, followed by time-frequency patterns of ERS and ERD, followed by statistically different (*p*FDR < 0.05) time frequency voxels between the two conditions. The last row shows the differences in EMG activity.

Time frequency analyses (ERSPs) considered activity within the 4–33 Hz bandwidth for the left and right μ clusters. The ERSP analyses show significant ERS/ERD changes from baseline across conditions in the left and right μ clusters. Figures 3–6 illustrate the results of time-frequency contrasts between the solo and each of the experimental speaking conditions. In addition to neural activity, activity from the EMG component is temporally decomposed so that neural activity can be temporally referenced to muscle activity.

#### Solo vs. Choral Speech

Figure 3 displays the solo vs. choral contrast. Significant differences found in choral speech are characterized by earlier and stronger μ-alpha and μ-beta ERD bilaterally. In the left hemisphere, choral speech produced stronger μ-alpha and μ-beta ERD before, during, and immediately after speech initiation. In the right hemisphere, choral speech produced stronger μ-alpha ERD across entire utterances. Additionally, differences in EMG activity were observed, with EMG activity being stronger in the choral condition prior to speech onset, but then becoming significantly weaker than the solo condition later in the utterance.

#### Solo vs. DAF

Figure 4 displays the solo vs. DAF contrast. Relative to the solo condition, there was significantly increased right hemisphere μ-alpha ERD in the DAF condition. No significant differences were observed in the left hemisphere. Additionally, EMG activity was weaker in the DAF condition.

#### Solo vs. Prolonged Speech

Figure 5 shows the solo vs. prolonged speech contrast. A few significant differences were observed between conditions in the time-frequency voxels, including about 700 ms of stronger right hemisphere μ-alpha ERD when prolonging syllables. However no clear pattern emerged that quantitatively differentiated between the conditions. Qualitatively, prolonged speech appears to produce more focal patterns of μ-alpha and μ-beta ERD, bilaterally.

#### Solo vs. Pseudostuttering

Figure 6 shows the solo vs. pseudostuttering contrast. Pseudostuttering produced significantly increased left hemisphere μ-beta ERD across utterances. While right hemisphere μ-alpha ERD also appeared stronger in pseudostuttering, no clear pattern of significant differences was observed in alpha frequencies relative to the control condition. EMG activity was relatively weaker in the pseudostuttering condition vs. the solo condition throughout speech production.

## Discussion

Left and right μ components were identified successfully from band-pass filtered concatenated EEG data during speech production conditions. 19/24 participants contributed components with ~5% unexplained RV. This proportion (79%) of useable components is similar to those found in previous studies (Nyström, 2008; Bowers et al., 2013; Jenson et al., 2014). It is important to note that in the current study, these proportions of contributors were achieved during complex, multi-syllable utterances using ICA to successfully separate neural from myogenic activity. Bilateral localization of components to the precentral gyrus across the premotor and sensorimotor cortices is consistent with accepted μ rhythm locations (Pineda, 2005; Hari, 2006) and sensorimotor function in speech production (Houde and Nagarajan, 2011; Tourville and Guenther, 2011). Time-frequency analyses then provided tests of the experimental hypothesis.

### Time Frequency Analyses

In the solo (control) condition, participants spoke in their normal unaltered manner. Oscillatory μ activity was characterized by bilateral μ-alpha/beta ERD. This pattern of desynchronization is consistent with previous findings in speech production (Jenson et al., 2014) and other movements including walking (Seeber et al., 2014; Wagner et al., 2016), reaching (Tan et al., 2014, 2016), swallowing (Dziewas et al., 2003; Cuellar et al., 2016), and tongue tapping (Cuellar et al., 2016). In the current study, ERSP power appears stronger in the left hemisphere and more diffuse in the right. This finding is important because it is consistent with left hemisphere dominance for sensorimotor control in speech production (Specht, 2014). By observing hemispheric differences in oscillatory activity, the data indicate that μ rhythm activity clearly is sensitive to sensorimotor contributions in speech production (Jenson et al., 2014, 2015). If hemispheric differences were not observed, it would suggest that μ-alpha and μ-beta were simply encoding primary somatosensory and motor responses, respectively.

#### Exogenous Experimental Conditions

Compared to the solo condition, the exogenous experimental conditions (e.g., choral speech and DAF) were characterized by increases in μ-alpha ERD in the right hemisphere throughout the utterance along with simultaneous decreases in EMG activity. It is worth pointing out that neither exogenous condition induced disfluency, which otherwise may have influenced neural activity. Instead, data strongly suggest that the change in μ-alpha activity is related to a change in sensorimotor control.

The finding that both conditions elicit increases in right hemisphere activity is interesting given the differences between the exogenous signals in each condition. One condition, DAF, is a straightforward manipulation of the speaker’s auditory feedback: a temporal delay in an auditory feedback signal elicits auditory error cell activity as well as responses to counteract the disrupting effects of DAF (e.g., decreases in speech rate; Katz and Lackner, 1977). These responses, in turn, are incorporated into the DAF and illustrate how an individual speaker can control aspects of the perturbation. The other condition, choral speech, does not alter the speaker’s auditory feedback but instead requires the speaker to co-produce speech utterances, more or less simultaneously, with an external auditory signal. Since the external signal is independent of the speaker, auditory feedback is evaluated with respect to an external signal or target on a moment-to-moment basis to ensure that they are broadly comparable. A similar mechanism has been proposed to account for the rapid and robust responses speakers exhibit when instructed to alter their pitch to follow the direction of an auditory perturbation (Hain et al., 2000; Patel et al., 2014).

Despite these differences, both exogenous conditions increase the engagement, or gain, of the auditory feedback controller compared to the solo speech condition. The finding of increased μ-alpha ERD in the right, but not left, hemisphere likely reflects changes in the contribution of feedforward and feedback controllers to speech output. Increases in right hemisphere activation have been associated with increases in feedback control in both speech and limb motor studies (Toyomura et al., 2007; Grafton et al., 2008; Tourville et al., 2008; Golfinopoulos et al., 2011). Future studies may examine the effect of longer delays more likely to induce disfluencies in neurotypical speakers (Stuart et al., 2002; Corey and Cuddapah, 2008). Data from the current study suggest that the presence of either exogenous signal was sufficient to increase sensorimotor processing in the right hemisphere consistent with a shift towards greater feedback control.

The right hemisphere changes in the alpha channel are also of methodological importance. The ubiquitous nature of alpha rhythms imbues them with sensitivity to a wide range of cognitive and motor functions across the sensorimotor cortex (Pfurtscheller and Berghold, 1989). However, changes in μ-alpha have been implicated in sensory feedback processing (Pfurtscheller and Berghold, 1989; Jenson et al., 2014, 2015). Increased μ-alpha ERD under exogenous conditions with an increased reliance on auditory feedback continue to support these notions.

Compared to the solo condition, the choral speech condition was characterized by bilateral increases in μ-alpha ERD and μ-beta ERD during speech initiation, consistent with stronger bilateral sensorimotor control during speech initiation in choral speech. These increases were observed early in the trial, prior to the cue to speak. This time period also was characterized by low level EMG activity. Though participants did not begin speaking until the cue, the early EMG activity may have been due to weak muscle contractions while listening to the carrier phrase, which may also have elicited μ ERD. Alternatively, early μ ERD may have been related to covert rehearsal of the carrier phrase or the text that was about to be read. Because overt and covert production both been shown to elicit μ-alpha ERD and μ-beta ERD (Jenson et al., 2014), it is currently not possible to separate these influences. However, the cumulative effect is the carrier phrase provided a “go signal” (Kilavik et al., 2013) that activated the sensorimotor system earlier in choral speech than in the solo condition. In other words, participants appeared to receive sensorimotor “priming” for speaking prior to receiving the choral signal. Advanced priming prior to speaking may have applications for treating initiation difficulties related to stuttering (Saltuklaroglu et al., 2009).

#### Endogenous Experimental Conditions

Compared to solo, in the left hemisphere the prolonged speech condition appeared to produce more focal patterns of μ-alpha ERD and μ-beta ERD in both hemispheres. However, consistent with findings from De Nil et al. (2008), no robust differences in μ rhythm oscillations were observed. In the right hemisphere, μ-alpha ERD appeared stronger in prolonged than in solo, suggesting that prolonged speech was characterized by increased feedback due to the novelty of the task and/or increased monitoring. However, this difference only achieved significance for about 700 ms of the 2000 ms analysis window. Decreased EMG activity suggested reduced articulatory movements, providing additional evidence that participants were elongating vowels as instructed.

In contrast to prolonged speech, the pseudostuttering condition was characterized by increased μ-beta ERD observed throughout utterances. This increase in sensorimotor activity suggests strong activation of forward models during prolonged speech and contrasts with findings of De Nil et al. (2008) who did not report changes in neural activity under a similar speaking condition. In the current study, increased μ-beta ERD was observed alongside decreased EMG activity. Reduced EMG activity may be due to the longer time necessary to produce pseudostuttered syllables which results in reduced speech output. However, the finding indicates that stronger μ-beta ERD is not directly related to movement and, therefore, reflects a change in sensorimotor control. Increased activation of forward modeling in the pseudostuttering condition may be a consequence of the same syllable being repeated, strengthening the sensory prediction. It may be similar to short-term motor learning, much like adaptation effects in stuttering (Max et al., 2004). This effect may also have been heightened by increased attention due to the novelty of the task.

Similar to the prolonged speech condition, pseudostuttering also produced increases in right hemisphere μ-alpha ERD, though they failed to reach significance. Taken together, the findings raise the possibility that all fluency enhancing conditions were characterized by increased auditory feedback control relative to the solo condition. However, the differences only achieved significance across speech events in the exogenous conditions, likely due to their additional auditory processing requirements. It is possible that feedback-related changes from endogenous conditions were not sufficient to elicit robust changes in sensorimotor control in NS populations or that the current study was underpowered for detecting those changes.

### Further Implications for Stuttering

Compared to the solo condition, exogenous and endogenous manipulations to speech motor control conditions produced differences in sensorimotor μ-alpha and μ-beta activity across the time course of utterances. In general, exogenous conditions in the current cohort of NS were associated with increased right hemisphere activity, likely reflecting increased feedback control (Grafton et al., 2008; Tourville et al., 2008). This may be important when applied to stuttering, as right hemisphere sensorimotor activity is thought to play a compensatory role for left hemisphere sensorimotor deficits (Sommer et al., 2002; Preibisch et al., 2003). However, more variable effects in PWS have been reported for exogenous signals, with choral speech and DAF producing decreases and increases in right hemisphere activity, respectively. Findings in PWS may be related to inherent variability noted with this disorder, levels of fluency achieved, or other influences such as scanner noise. As such, further testing using these quiet, temporally sensitive measures in PWS will better explain how fluency enhancing conditions work in PWS and whether they differentially alter sensorimotor control in PWS vs. NS.

Endogenous conditions were associated with relatively fewer changes in sensorimotor control, though pseudostuttering produced stronger left hemisphere sensorimotor activations, suggesting it may produce increases in left-hemisphere based feedforward control. Data may be compared to those from PWS who exhibit both long- (De Nil et al., 2003; Neumann et al., 2005) and short- (De Nil et al., 2008) term differences in sensorimotor control as a result of using endogenous techniques. The findings in PWS suggest that endogenous techniques may alter the relative contributions of both feedforward and feedback control, though further investigation is necessary. It also is interesting to consider how other endogenous fluency enhancing motor manipulations, such as whispering (Bloodstein, 1950) and accent modification (Bloodstein, 1950) might alter sensorimotor control in both PWS and neurotypical populations.

### Limitations

Though all participants produced “μ-like” components, some did not meet inclusion criteria. This is common in oscillatory studies due to inherent inter-individual variability when mapping neural function to cortical topography (Biermann-Ruben et al., 2005; Basile, 2007). The main reason some components did not meet inclusion criteria was due to noisy spectra. Despite filtering and ICA, which removed stereotypical noise, there was still a considerable amount of non-stereotypical noise present in the data, possibly due to the length and complexity of the spoken utterances. Additional causes for not being included in μ clusters are: (1) ICA failed to assign what appeared to be a μ component to one of the cortical regions known to generate sensorimotor μ rhythms; and (2) ICA failed to fit a μ component dipole in the sensorimotor region with less than 20% RV. These problems likely stem from the use of standard head models and channel locations, which reduce source localization accuracy. Such limitations continue to highlight the general need for improved spatial resolution in EEG techniques. Also, while the results indicate that participants complied with instructions in all speaking conditions, this assertion would be bolstered by additional acoustic and/or kinematic data.

### Conclusions and Future Directions

ICA successfully unmixed raw EEG data collected during speech production to identify sensorimotor μ components. Subsequent time-frequency analyses of μ component clusters revealed oscillatory changes in μ-alpha and μ-beta bands which were indicative of real-time adjustments in sensorimotor control that were differentiated by speaking condition. The data provide further evidence that the temporal resolution of EEG can be exploited for understanding sensorimotor control in NS populations with an eye towards examining differences in sensorimotor control related to stuttering and other communication disorders. A number of recent studies continues to demonstrate the utility of EEG for examining sensorimotor differences related to stuttering. For example, Daliri and Max (2018) used measures of N1 amplitude during speech motor planning to demonstrate that DAF reduced auditory modulations in NS but increased them in PWS. Sengupta et al. (2016) demonstrated that PWS showed reduced sensorimotor adaptation to DAF that was accompanied by regional differences in EEG phase coherence. Using a similar paradigm to this one, Saltuklaroglu et al. (2017) demonstrated reduced μ-beta spectral amplitudes and differences in PWS, accompanied by stuttering-related μ-alpha and μ-beta oscillatory differences in speech and tone perception tasks. To better inform regarding the neurophysiology and amelioration of stuttering, future studies using these techniques should extend to examining measures of μ and auditory connectivity differences between PWS and NS, characterizing the timing and the directionality of auditory-motor interactions before, during, and after the utterance. In addition to helping elucidate sensorimotor differences related to stuttering, such data will help inform current theories and models of sensorimotor control in normal speech production.

## Author Contributions

TK and TS conceived and designed the study, performed the statistical analyses, wrote the first version of the manuscript, and edited all subsequent versions. DT and DEJ assisted TK with data collection and analysis. TK, KJR, TS, AWH, DC, TH and ALB wrote and/or edited sections of the manuscript. All authors contributed to manuscript revision, read and approved the submitted version.

## Conflict of Interest Statement

The authors declare that the research was conducted in the absence of any commercial or financial relationships that could be construed as a potential conflict of interest.
